# Tirzepatide in Obesity-Related Obstructive Sleep Apnea: Beyond Weight Loss Toward Disease Modification?

**DOI:** 10.3390/life16050802

**Published:** 2026-05-12

**Authors:** Florin-Dumitru Mihălțan, Corina Ioana Borcea, Ancuța Alina Constantin

**Affiliations:** 1 Department of Cardio-Thoracic Pathology, “Carol Davila” University of Medicine and Pharmacy, 050474 Bucharest, Romania; florin.mihaltan@umfcd.ro; 2 Institute of Pneumology “Marius Nasta”, 050159 Bucharest, Romania; corina.borcea@umfcd.ro

**Keywords:** obesity, obstructive sleep apnea, tirzepatide, GLP-1/GIP receptor agonists, cardiometabolic risk, public health burden

## Abstract

Background: Obesity is a major driver of obstructive sleep apnea (OSA), traditionally managed as a mechanical disorder of upper airway collapse. However, growing evidence supports a broader pathophysiological model involving metabolic dysfunction, systemic inflammation, and ventilatory instability. Tirzepatide, a dual glucose-dependent insulinotropic polypeptide (GIP) and glucagon-like peptide-1 (GLP-1) receptor agonist, has demonstrated substantial weight loss and cardiometabolic benefits, raising the possibility of a paradigm shift in OSA management. Objective: To critically evaluate whether tirzepatide may act as a disease-modifying therapy in obesity-related OSA beyond its effects on weight reduction. Methods: A narrative review was conducted using PubMed, Scopus, and Web of Science up to January 2026. Evidence from randomized controlled trials, meta-analyses, and mechanistic studies on incretin-based therapies in obesity and OSA was synthesized, with emphasis on clinical outcomes and underlying biological pathways. Results: Tirzepatide is associated with significant reductions in apnea–hypopnea index (AHI), accompanied by substantial weight loss. However, emerging data suggest that improvements in OSA severity may not be entirely explained by weight reduction alone. Potential weight-independent mechanisms include modulation of systemic inflammation, improvements in insulin sensitivity, alterations in adipokine profiles, and effects on autonomic regulation and ventilatory control. These pleiotropic effects may influence key components of OSA pathophysiology, including upper airway stability and chemosensitivity. Despite these findings, current evidence remains insufficient to definitively distinguish weight-dependent from weight-independent effects. Conclusions: Tirzepatide represents a promising therapeutic advance in obesity-related OSA, with potential implications extending beyond weight loss toward disease modification. While current data support a substantial role in reducing OSA severity, definitive confirmation of disease-modifying effects requires further mechanistic and long-term clinical studies. This emerging paradigm points to a shift from purely device-based management toward integrated, pathophysiology-driven treatment strategies.

## 1. Introduction

Obesity is a chronic, relapsing disease that requires lifelong, multidisciplinary management, including behavioral, pharmacological, and surgical interventions, alongside the prevention and treatment of associated comorbidities. Its global burden has increased substantially, with prevalence more than doubling in adults and quadrupling in adolescents since 1990. In 2022, approximately 890 million adults were living with obesity and 2.5 billion were overweight, representing 16% and 43% of the global adult population, respectively. The burden extends to younger populations, with over 390 million children and adolescents and 35 million children under five affected [1,2]. In the United States, obesity affects approximately one-third of the population and contributes substantially to disease burden and mortality, with healthcare costs projected to reach $3 trillion annually by 2030 [3,4,5].

Among its major complications, obesity is closely associated with sleep-disordered breathing, particularly OSA, which affects more than one billion individuals worldwide. Prevalence estimates range from 4% to 30% in the general population, with meta-analyses reporting rates between 9% and 38%, increasing with age and more common in men [6,7]. Obesity–hypoventilation syndrome (OHS) represents a severe obesity-related respiratory condition, with OSA present in approximately 90% of cases and severe OSA in nearly 70% of patients [8,9]. Although relatively uncommon in the general population (0.15–0.3%), OHS is significantly more prevalent among individuals with obesity and OSA, affecting up to 20% of these patients [9,10,11].

Despite advances in positive airway pressure (PAP) therapy, OSA continues to be primarily managed as a mechanical disorder characterized by upper airway collapse, rather than as a systemic condition involving metabolic and inflammatory dysfunction [6,12]. While PAP effectively alleviates airway obstruction, it does not address key underlying contributors such as obesity, systemic inflammation, and cardiometabolic dysregulation [13].

Recent developments in incretin-based therapies, particularly the dual GIP and GLP-1 receptor agonist tirzepatide, represent an emerging therapeutic approach with the potential to modify current management strategies in obesity-related OSA [14]. Clinical trials have demonstrated significant reductions in AHI, largely attributed to substantial weight loss [15,16]. However, emerging evidence suggests additional mechanisms—including modulation of inflammatory pathways, autonomic function, and metabolic homeostasis—may also contribute to improvements in OSA severity [12,17,18]. Importantly, the relative contribution of weight-dependent versus weight-independent mechanisms remains unclear.

These observations raise a key unresolved question: does tirzepatide act solely as a weight-reduction agent in OSA, or could it represent a disease-modifying therapy targeting the underlying pathophysiology of obesity-related sleep-disordered breathing [13,19]? Accordingly, this review aims to critically evaluate whether tirzepatide may act as a disease-modifying therapy in obesity-related OSA, with particular emphasis on mechanisms beyond weight loss and implications for future therapeutic strategies.

## 2. Literature Search Strategy

This narrative review is based on a comprehensive and systematic literature search conducted across PubMed, Scopus, and Web of Science, covering publications up to January 2026. The search strategy incorporated combinations of the following keywords: “tirzepatide,” “GLP-1 receptor agonists,” “GIP,” “obstructive sleep apnea,” “OSA,” “obesity,” “apnea–hypopnea index,” and “incretin-based therapy.”

Studies were considered eligible if they involved human participants and reported outcomes related to obstructive sleep apnea, particularly the apnea–hypopnea index (AHI), or investigated mechanistic pathways linking metabolic dysfunction with respiratory physiology. Particular emphasis was placed on randomized controlled trials, meta-analyses, and high-quality observational studies assessing the impact of incretin-based therapies on obesity and OSA-related outcomes. In addition, preclinical and mechanistic studies were included to provide complementary insights into underlying pathophysiological processes, including inflammation, autonomic regulation, and ventilatory control.

Exclusion criteria comprised non-peer-reviewed publications, studies lacking sleep-related outcomes, and articles not available in English. The screening process was conducted independently by two reviewers, initially through title and abstract assessment, followed by full-text evaluation of potentially relevant studies. Any discrepancies were resolved through consensus.

To ensure comprehensiveness, the reference lists of selected articles were also examined to identify additional relevant studies. The final selection was guided by relevance to the research topic, methodological rigor, and recency of publication, with particular emphasis placed on studies published within the past five years.

## 3. History of Tirzepatide

Pharmacological interventions have become an important component in the management of obesity, particularly in individuals who do not achieve adequate weight loss through lifestyle modification alone. Tirzepatide was approved by the U.S. Food and Drug Administration in 2022 as an adjunct to diet and exercise for improving glycemic control in patients with type 2 diabetes mellitus, and in 2023 for chronic weight management in adults with obesity or overweight with at least one weight-related comorbidity [20].

Among emerging pharmacological treatments, tirzepatide—a dual GLP-1 and GIP receptor agonist—has demonstrated superior efficacy in weight reduction compared with existing therapies [21]. This reflects the ongoing evolution of obesity pharmacotherapy and highlights the potential for more effective treatments with favorable safety profiles.

Tirzepatide improves glycemic control and induces significant weight loss in patients with type 2 diabetes, with effects comparable or superior to established GLP-1 receptor agonists such as semaglutide. It is administered once weekly via subcutaneous injection, with dose escalation based on tolerability. Clinical trials have shown a clear dose–response relationship, although the extent of weight reduction may be limited by gastrointestinal adverse effects at higher doses.

In clinical practice, tirzepatide is increasingly used within multidisciplinary obesity management programs, often alongside lifestyle interventions and specialist support. Its high efficacy has contributed to growing interest among clinicians and patients, particularly in the context of expanding pharmacological options for obesity treatment.

## 4. Mechanism of Action

Tirzepatide is a synthetic polypeptide that acts as a dual GLP-1 and GIP receptor agonist. As a 39-amino-acid peptide and analog of gastric inhibitory polypeptide, it exhibits distinct pharmacological properties compared with selective GLP-1 receptor agonists. Functionally, tirzepatide enhances insulin secretion, reduces hyperglycemia, and modulates metabolic pathways involved in glucose and lipid homeostasis.

Signaling studies have shown that tirzepatide mimics the actions of native GIP at the GIP receptor, while at the GLP-1 receptor, it demonstrates biased agonism toward cyclic AMP (cAMP) generation rather than β-arrestin recruitment [22]. This dual receptor activity and signaling profile are thought to enhance insulin secretion and metabolic regulation [14].

Tirzepatide is associated with significant changes in adipokine profiles, including increases in adiponectin—an important regulator of glucose and lipid metabolism—with reported increases of up to 26% after 26 weeks at a 10 mg dose [23]. In parallel, reductions in leptin levels have been observed, reflecting improvements in adipose tissue function and metabolic status [24]. Incretin-based therapies may also influence autonomic regulation; GLP-1 receptor agonists have demonstrated effects on the carotid body, potentially attenuating hyperglycemia-induced sympathetic activation [17].

The dual agonist mechanism of tirzepatide results in greater reductions in hyperglycemia and appetite compared with GLP-1 receptor agonists alone. In individuals without diabetes, treatment with tirzepatide (5–15 mg once weekly) has been associated with substantial weight loss, ranging from 16.5% to 22.4% over 72 weeks, alongside improvements in insulin sensitivity and β-cell function [23,25]. As the first dual GIP and GLP-1 receptor agonist, tirzepatide represents a new class of agents with enhanced efficacy in weight reduction and metabolic control [26].

Beyond glycemic control, GLP-1 receptor agonists exert cardiovascular protective properties, including reductions in oxidative stress, inflammation, endoplasmic reticulum stress, apoptosis, and adverse vascular remodeling, contributing to improved endothelial function and cardiovascular outcomes [27].

Taken together, these mechanisms provide a biologically plausible framework linking incretin-based therapy to multiple dimensions of OSA pathophysiology beyond weight reduction alone.

## 5. Pharmacokinetics

Tirzepatide is an analog of the human GIP hormone, modified by the addition of a C20 fatty diacid moiety to optimize its pharmacokinetic profile [28]. This fatty-diacid component (eicosanedioic acid) is linked via a glutamic acid spacer and additional linker units to the lysine residue, enhancing albumin binding and thereby prolonging its half-life [29]. Following subcutaneous administration, tirzepatide has an estimated bioavailability of approximately 80%, with peak serum concentrations reached within 8 to 72 h. It exhibits a mean steady-state volume of distribution of approximately 10.3 L and is highly bound to plasma albumin (approximately 99%).

Tirzepatide undergoes proteolytic cleavage of its peptide backbone, while the fatty-diacid component is metabolized through β-oxidation and amide hydrolysis. As a modified polypeptide, it is ultimately degraded into individual amino acids in multiple tissues, including the liver [30]. The elimination half-life is approximately 5 days, supporting once-weekly dosing, with excretion occurring via both urine and feces in the form of metabolites [31].

From a clinical perspective, these pharmacokinetic properties enable sustained receptor activation and facilitate adherence through once-weekly administration. In the context of obstructive sleep apnea, this profile is particularly relevant, as it supports long-term weight reduction and metabolic stabilization—key determinants of OSA severity and progression.

## 6. Benefits of Tirzepatide: Study Results

### 6.1. Clinical Effects of Tirzepatide in OSA

OSA is associated with activation of inflammatory pathways, including inflammasome activation in monocytes and macrophages, which contribute to its neurocognitive and cardiovascular complications. Both continuous positive airway pressure (CPAP) and incretin-based therapies, such as glucagon-like peptide-1 receptor agonists (GLP-1RAs), may modulate these inflammatory processes, suggesting overlapping mechanistic effects [32].

Incretin-based therapies, including GLP-1 and dual GLP-1/GIP receptor agonist, have demonstrated promising results in obesity management and have therefore generated increasing interest in the treatment of OSA [15]. A systematic review and meta-analysis by Bardóczi et al. identified five randomized controlled trials (*n* = 1024) evaluating incretin-based therapies in patients with OSA over a duration of at least 12 weeks. These therapies were associated with a significant reduction in AHI, with a mean change of −14.45 events/h (95% CI: −25.90 to −2.99; *p* < 0.001), and demonstrated greater efficacy compared with usual care (mean difference −11.61 events/h; 95% CI: −22.91 to −0.31; *p* = 0.046) [15].

In two randomized, double-blind, placebo-controlled trials, double-blind, placebo-controlled trials including 469 patients with moderate-to-severe OSA, tirzepatide significantly reduced AHI compared with placebo, both in patients treated with and without PAP therapy. At baseline, AHI values of approximately 50 events/h, reductions of 25.3 and 20.0 events/h were observed, respectively. Notably, up to 50.2% of participants achieved either an AHI < 5 events/h or an AHI of 5–14 events/h with an Epworth Sleepiness Scale score ≤ 10, a threshold at which PAP therapy may no longer be indicated [33].

Across studies, tirzepatide treatment is associated with reductions in AHI of approximately 20–24 events/h at 52 weeks, accompanied by 16–17% weight loss and improvements in OSA-specific hypoxic burden and systemic inflammation, as reflected by reductions in high-sensitivity C-reactive protein levels [15,33,34]. In patients unable or unwilling to use PAP therapy, incretin-based therapies may provide clinically meaningful improvements in OSA severity and related symptoms [15].

The SURMOUNT-OSA program, consisting of two randomized, placebo-controlled trials evaluating tirzepatide (10 mg or 15 mg) over 52 weeks in patients with obesity and moderate-to-severe OSA, further demonstrated significant improvements in sleep-related outcomes, including sleep disturbance, functional status, and health-related quality of life [35]. However, these studies did not include direct head-to-head comparisons with PAP therapy, leaving an important gap in comparative effectiveness. Nevertheless, the magnitude of weight loss observed was associated with reductions in OSA severity and sleepiness that may approach those achieved with PAP, while also providing additional benefits in cardiometabolic risk factors such as blood pressure [19].

The recent approval of tirzepatide as a pharmacological treatment for obesity-related OSA represents a significant advancement in the field. Combination or multimodal approaches, including pharmacological therapy alongside PAP or lifestyle interventions, may offer greater efficacy than monotherapy [14]. Real-world evidence from large cohorts of patients with OSA and obesity suggests initiation of tirzepatide is associated with reduced risks of all-cause mortality, major adverse cardiovascular events, and adverse kidney outcomes compared with lifestyle interventions alone [36,37].

While these findings support a substantial role for tirzepatide in reducing OSA severity, it remains unclear to what extent these improvements are mediated exclusively by weight loss versus additional metabolic and neurophysiological mechanisms. This distinction is clinically important, as it may determine whether tirzepatide should be considered primarily as an adjunctive therapy or as a potential disease-modifying intervention in selected patients. The main clinical trials evaluating tirzepatide in obesity and obstructive sleep apnea are summarized in Table 1, illustrating consistent improvements in weight and apnea–hypopnea index, while underscoring the need to further clarify weight-dependent and weight-independent mechanisms.

Overall, these findings demonstrate consistent benefits of tirzepatide across clinical and real-world settings.

### 6.2. Tirzepatide, OSA and Diabetes

OSA is highly prevalent among individuals with obesity and type 2 diabetes mellitus (T2DM), with reported rates of OSA (defined as an apnea–hypopnea index > 5 events/h) reaching up to 86.6% in this population [42]. This close association reflects shared pathophysiological mechanisms, including insulin resistance, systemic inflammation, and altered neurohumoral regulation, which contribute to both metabolic dysfunction and respiratory instability.

Tirzepatide, a recently approved therapy for T2DM, has demonstrated substantial efficacy in improving glycemic control and inducing significant weight reduction [43,44]. In clinical trials involving individuals with obesity without diabetes, once-weekly administration of tirzepatide resulted in mean weight reductions of up to 20.9% at the highest evaluated dose (15 mg), compared with 3.1% in the placebo group [38]. These effects are particularly relevant in OSA, where excess adiposity and insulin resistance play a central role in disease severity.

In addition to weight reduction, both tirzepatide and semaglutide have been associated with significant reductions in the long-term risk of developing T2DM, with relative risk reductions of approximately 60–69% over 10 years [18]. Given the bidirectional relationship between OSA and metabolic dysfunction, improvements in glycemic control may reduce the overall cardiometabolic burden.

Among patients with established T2DM, tirzepatide has demonstrated significant improvements in glycated hemoglobin levels, with reductions ranging from 1.87% to 3.02% across clinical trials [45]. In patients with coexisting OSA, these metabolic benefits may translate into improved disease control, particularly when combined with standard therapies such as CPAP [46].

Overall, the close interplay between OSA, obesity, and T2DM suggests that therapies targeting metabolic dysfunction may have a broader impact on disease expression. Tirzepatide, by addressing both weight and glycemic control, may therefore represent an important component of integrated treatment strategies in patients with OSA and metabolic comorbidities, although OSA-specific outcomes remain insufficiently studied.

### 6.3. Tirzepatide and Obesity: Implications for OSA

Obesity is the principal modifiable risk factor for obstructive sleep apnea (OSA), contributing to upper airway narrowing, reduced neuromuscular control, and increased collapsibility during sleep [6,12]. Excess adiposity, particularly in the visceral and upper airway regions, plays a central role in disease pathogenesis, while weight reduction remains one of the most effective non-device-based strategies for improving OSA severity.

Tirzepatide has demonstrated substantial efficacy in inducing weight loss, with clinical trials reporting mean reductions of approximately 20% of baseline body weight. In the SURMOUNT-1 trial, treatment with tirzepatide resulted in mean weight reductions of 19.5% and 20.9% at the 10 mg and 15 mg doses, respectively, compared with 3.1% in the placebo group at 72 weeks [38,47]. A large proportion of participants achieved clinically meaningful weight loss thresholds, with more than 60% attaining ≥15% weight reduction and over half achieving ≥20%, a magnitude comparable to that observed with bariatric surgery.

In the context of OSA, weight reduction of this magnitude is strongly associated with improvements in apnea–hypopnea index (AHI), upper airway function, and cardiometabolic risk [16]. However, the relationship between weight loss and OSA improvement is not strictly linear, and residual disease often persists despite substantial weight reduction, suggesting that additional mechanisms may contribute to treatment response.

The durability of weight loss is also a critical consideration. Studies have shown that individuals may regain a substantial proportion of lost weight following discontinuation of tirzepatide therapy, with estimates exceeding 50% within one year [48,49]. In patients with OSA, such weight regain may lead to recurrence or worsening of disease severity, highlighting the importance of sustained treatment strategies.

Adjunctive approaches may further enhance outcomes. The SURMOUNT-3 trial demonstrated the efficacy of tirzepatide following intensive lifestyle intervention, supporting its role as part of a comprehensive, multidisciplinary approach to obesity management [50]. Given the multifactorial nature of OSA, combining pharmacological therapy with behavioral and device-based interventions may offer additive or synergistic benefits.

Beyond weight reduction, tirzepatide is associated with improvements in metabolic parameters, including insulin sensitivity and lipid profiles, which are closely linked to OSA-related cardiometabolic risk [18]. These effects may contribute to a broader reduction in disease burden, extending beyond improvements in respiratory parameters alone.

Patient-reported outcomes also suggest meaningful clinical benefits with incretin-based therapies. Individuals receiving GLP-1 receptor agonists have reported improvements in overall health status and functional capacity, with more favorable outcomes observed among those receiving longer durations of therapy [51].

Overall, while weight loss remains the dominant mechanism through which tirzepatide improves OSA, the magnitude of its effects and associated metabolic changes suggest a more complex interaction with disease pathophysiology. This reinforces the potential role of tirzepatide as part of an integrated therapeutic strategy targeting both obesity and OSA.

### 6.4. Tirzepatide and Cardiovascular Diseases

OSA is strongly associated with increased cardiovascular morbidity and mortality, driven by intermittent hypoxia, sympathetic overactivation, systemic inflammation, and metabolic dysregulation [6,12]. These mechanisms contribute to the development of hypertension, atherosclerosis, and heart failure, highlighting the need for therapeutic strategies that address both respiratory and cardiometabolic pathways. Tirzepatide has demonstrated consistent improvements in multiple cardiometabolic parameters across the SURPASS clinical trial program, including reductions in visceral adiposity, improved insulin sensitivity, and attenuation of myocardial lipotoxicity [52,53,54]. These effects are particularly relevant in OSA, where visceral fat accumulation and insulin resistance contribute to both upper airway instability and cardiovascular risk. By targeting these mechanisms, tirzepatide may indirectly reduce the cardiovascular burden associated with OSA.

Incretin-based therapies have also been shown to exert pleiotropic cardiovascular effects beyond glycemic control, including reductions in oxidative stress, inflammation, and endothelial dysfunction [27,55]. These pathways are central to OSA pathophysiology, where recurrent hypoxia–reoxygenation cycles promote vascular injury and atherogenesis. Accordingly, modulation of these processes may contribute to improved vascular function in patients with OSA.

Tirzepatide has been associated with clinically meaningful reductions in blood pressure, including decreases of approximately 11 mmHg systolic and 5.6 mmHg diastolic in clinical studies [56]. This is particularly relevant in OSA, where hypertension is frequently resistant to treatment and driven by sustained sympathetic activation. Improvements in blood pressure control may therefore represent an important mechanism linking metabolic therapy to cardiovascular risk reduction in this population.

Emerging evidence further supports beneficial effects on cardiac structure and function. Tirzepatide has been associated with improvements in myocardial performance and reductions in adverse remodeling, mediated in part by improvements in metabolic and inflammatory pathways [53,57,58]. In addition, a phase III trial demonstrated clinically meaningful benefits in patients with obesity and heart failure with preserved ejection fraction (HFpEF) [59], a condition highly prevalent among individuals with OSA.

Cardiovascular safety has been demonstrated in the SURPASS-4 trial, in which tirzepatide showed a favorable risk profile compared with insulin glargine, with a hazard ratio of 0.74 (95% CI: 0.51–1.08) for major adverse cardiovascular events [60]. Additional analyses suggest a reduction in the relative risk of cardiovascular death or worsening heart failure, although event numbers remain limited [59,61].

While these findings support a potential role for tirzepatide in reducing cardiovascular risk, most evidence derives from populations without OSA. Therefore, although highly relevant to OSA pathophysiology, dedicated studies in OSA-specific cohorts are required to define its clinical impact in this setting.

### 6.5. Effects on Traditional CV Risk Factors

Most randomized controlled trials evaluating incretin-based therapies have reported significant improvements in conventional cardiovascular risk factors, including lipid profiles and blood pressure. Tirzepatide demonstrates similar benefits [18]. In pooled analyses, incretin-based therapies have been associated with increases in high-density lipoprotein (HDL) cholesterol of up to approximately 13%, with the exception of orfoglipron.

In patients with type 2 diabetes, tirzepatide has also been shown to reduce albuminuria and significantly slow the decline in estimated glomerular filtration rate (eGFR) compared with insulin glargine [62]. In the SURMOUNT-1 trial, a phase 3 randomized study evaluating tirzepatide in individuals with overweight or obesity without diabetes, treatment was associated with significant placebo-adjusted improvements in systolic and diastolic blood pressure, triglyceride levels, and waist circumference after 72 weeks [38].

### 6.6. Comparison with Other Molecules

The SURMOUNT-5 trial, recently published in the New England Journal of Medicine, demonstrated that tirzepatide was associated with greater reductions in body weight and waist circumference over approximately 72 weeks compared with semaglutide [41].

Consistent with these findings, earlier studies have shown that tirzepatide produces greater weight loss than other incretin-based therapies. In the SURMOUNT-1 trial, tirzepatide achieved a placebo-adjusted reduction in body weight of approximately 18%, exceeding the effects observed with liraglutide in previous studies [38]. Given the established relationship between weight reduction and improvements in OSA severity, these findings support the potential clinical relevance of tirzepatide in this population. However, it has also been suggested that weight reductions beyond 20% may confer only limited additional improvements in AHI [16].

In patients with type 2 diabetes mellitus, tirzepatide has demonstrated superior glycemic control and weight reduction compared with several agents, including semaglutide, dulaglutide, insulin degludec, and insulin glargine, as reported in the SURPASS clinical trial program [39,40]. Comparative analyses further support these findings, showing greater percentage body weight reduction with tirzepatide 15 mg compared with semaglutide 2.4 mg and liraglutide 3.0 mg [63].

Pooled and meta-analytic data also indicate that tirzepatide is associated with significantly greater weight reduction compared with semaglutide, with higher odds of achieving clinically meaningful weight loss (≥10%) [64]. A network meta-analysis including multiple doses of tirzepatide and semaglutide in non-diabetic individuals with obesity demonstrated that tirzepatide at higher doses provides the greatest efficacy in weight reduction, although this benefit is accompanied by increased gastrointestinal adverse events and higher discontinuation rates [65].

These findings highlight a clear dose–response relationship for tirzepatide and underscore the importance of balancing efficacy with tolerability when selecting therapy. They also emphasize the need for individualized treatment strategies based on patient characteristics, therapeutic goals, and tolerance to incretin-based therapies.

### 6.7. Tirzepatide Versus Bariatric Surgery: Advantages, Limitations, and Current Comparative Evidence

Bariatric surgery remains the best-established weight-loss intervention for severe obesity and has consistently been associated with improvement in obstructive sleep apnea (OSA). Systematic reviews and meta-analyses have shown that bariatric surgery reduces body mass index and apnea–hypopnea index (AHI), although complete remission is not universal and OSA persists in many patients at follow-up [66,67]. More recent pooled evidence likewise supports significant improvement in OSA severity after bariatric surgery, but with heterogeneity across procedures, baseline obesity severity, and duration of follow-up [68].

By contrast, tirzepatide offers a non-surgical treatment option that is reversible and easier to integrate into multidisciplinary obesity care. In the phase 3 SURMOUNT-OSA trials, tirzepatide significantly reduced AHI, body weight, hypoxic burden, high-sensitivity C-reactive protein, and systolic blood pressure, while also improving sleep-related patient-reported outcomes in adults with obesity and moderate-to-severe OSA [33]. These features make tirzepatide particularly attractive for patients who decline surgery, are poor surgical candidates, or prefer a less invasive initial strategy.

From a practical perspective, bariatric surgery may offer greater long-term weight-loss durability, but it is invasive and carries perioperative risk, nutritional consequences, and the need for long-term postoperative monitoring [69]. Tirzepatide is less invasive and avoids surgical morbidity, but it requires chronic treatment, may be limited by gastrointestinal adverse effects, and raises concerns regarding weight regain after discontinuation [33,69].

Direct comparative evidence between tirzepatide and bariatric surgery in OSA is currently very limited. At present, no randomized head-to-head trial and no formal non-inferiority trial have established that tirzepatide is non-inferior to bariatric surgery for OSA-specific outcomes. The main comparative study available is a 2025 multi-institutional propensity score-matched retrospective analysis, which found similar overall composite clinical outcomes between tirzepatide and bariatric metabolic surgery in adults with OSA and obesity, although tirzepatide was associated with fewer kidney-related complications [70]. However, this study did not constitute a non-inferiority trial and did not directly compare detailed polysomnographic end points such as AHI change or OSA remission.

Overall, current evidence supports tirzepatide as a promising alternative or adjunct to bariatric surgery in selected patients with obesity-related OSA, but not yet as a proven non-inferior replacement. Bariatric surgery remains the more established intervention for durable weight loss and OSA improvement, whereas tirzepatide offers a less invasive option with strong randomized evidence versus placebo, but not yet versus surgery [33,66,67,68,69,70].

### 6.8. Economic Considerations: Cost of Tirzepatide and Potential Impact on OSA-Related Healthcare Burden

The cost of tirzepatide represents a major consideration for its implementation in clinical practice. In the United States, recent pricing data indicate that monthly costs for tirzepatide (Zepbound) range approximately between $300 and $450 in manufacturer-supported self-pay programs, with higher effective costs depending on insurance coverage and negotiated pricing [71]. However, broader health-economic analyses suggest annual net prices of several thousand dollars per patient, reflecting the high budget impact of long-term treatment [72].

From a cost-effectiveness perspective, modeling studies evaluating tirzepatide for obesity management have reported incremental cost-effectiveness ratios within commonly accepted thresholds, although results are highly sensitive to drug pricing and long-term adherence [73]. Some analyses suggest that tirzepatide may approach cost-effectiveness under specific pricing scenarios or healthcare systems, but widespread use remains constrained by overall budget impact due to the large eligible population [72].

OSA is associated with substantial direct and indirect costs. These include increased healthcare utilization, cardiovascular morbidity, and reduced productivity related to daytime sleepiness and accident risk. Weight reduction has been consistently associated with reductions in healthcare costs, with economic analyses demonstrating that improvements in obesity-related outcomes can translate into measurable savings at the population level [74].

Despite the demonstrated efficacy of tirzepatide in reducing body weight and improving OSA severity, direct evidence evaluating its cost-effectiveness specifically in patients with OSA is currently lacking. Existing economic models primarily focus on obesity and cardiometabolic outcomes, incorporating OSA only as a secondary complication rather than a primary endpoint [72,73].

Therefore, while tirzepatide has the potential to reduce both direct and indirect costs associated with OSA through improvements in weight, metabolic health, and disease severity, this remains a theoretical and indirect benefit. Dedicated cost-effectiveness analyses in OSA populations, incorporating sleep-specific outcomes, healthcare utilization, and productivity measures, are needed to confirm its economic value.

## 7. Is Tirzepatide a Disease-Modifying Therapy in OSA?

The therapeutic effects of tirzepatide in OSA have been largely attributed to its profound impact on body weight reduction [15,16]. However, the magnitude of improvement in AHI and hypoxic burden observed in clinical trials raises the possibility that additional mechanisms may be involved [19,33].

Obesity contributes to OSA not only through mechanical loading of the upper airway but also via systemic inflammation, altered ventilatory control, and neurohumoral dysregulation [6,12]. Tirzepatide has been shown to reduce inflammatory markers, improve insulin sensitivity, and modulate adipokine profiles, including increases in adiponectin and reductions in leptin levels [18,23,24]. These effects may influence respiratory control stability, upper airway neuromuscular function, and chemosensitivity.

**Figure 1:**
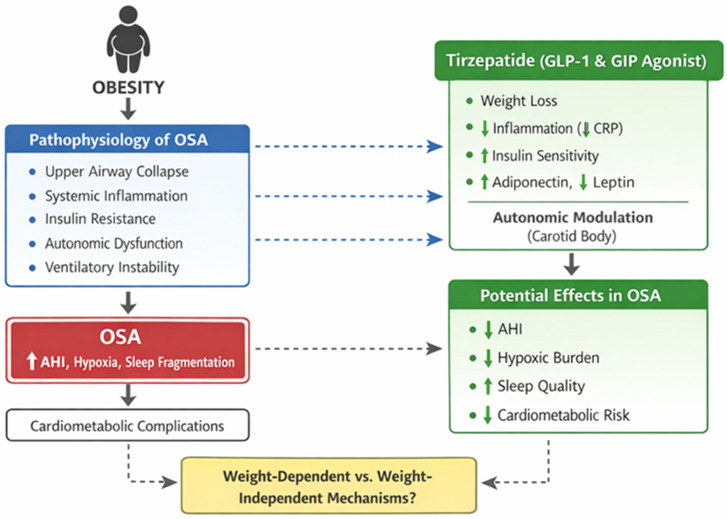
Proposed mechanisms of tirzepatide in obesity-related obstructive sleep apnea. (Blue boxes/arrows represent obesity-related OSA pathophysiology; green boxes/arrows represent tirzepatide actions and potential therapeutic effects; the red box highlights OSA clinical consequences; the yellow box indicates the unresolved distinction between weight-dependent and weight-independent mechanisms. Dashed arrows indicate proposed or indirect mechanistic pathways).

Furthermore, GLP-1 receptor agonists have demonstrated actions at the level of the carotid body and autonomic nervous system, suggesting a potential role in modulating ventilatory responses to hypoxia and hypercapnia (Figure 1) [17]. Although direct evidence for tirzepatide in this context remains limited, these pathways provide a plausible biological framework for effects extending beyond weight loss alone.

Importantly, the relationship between weight reduction and AHI improvement is not strictly linear, and some patients experience disproportionate benefits in OSA severity relative to weight change [16]. This observation supports the hypothesis that incretin-based therapies may exert disease-modifying effects by targeting multiple components of OSA pathophysiology.

Nevertheless, current evidence is insufficient to definitively separate weight-dependent from weight-independent effects. Future studies incorporating mechanistic endpoints—such as ventilatory control parameters, upper airway collapsibility, and inflammatory biomarkers—are essential to clarify whether tirzepatide represents a true disease-modifying therapy in OSA or primarily a highly effective adjunct through weight reduction [13,19].

## 8. Adverse Effects and Safety Considerations

Tirzepatide has been shown to exert anti-inflammatory effects in preclinical models, including inhibition of aeroallergen-induced airway inflammation in a mouse model of asthma [75]. In clinical studies involving individuals with obesity without T2DM, tirzepatide has been associated with a higher incidence of gastrointestinal adverse events compared with placebo, including nausea, vomiting, diarrhea, decreased appetite, eructation, and dyspepsia [76].

Safety analyses indicate no significant increase in the relative risk of pancreatitis, although an elevated risk of biliary disease has been reported (relative risk 1.97) [77]. Acute kidney injury may occur, primarily secondary to dehydration and volume depletion, particularly in the context of gastrointestinal side effects [78]. Hypoglycemic events are uncommon but may occur more frequently in patients receiving concomitant sulfonylurea therapy [79]. Tirzepatide is contraindicated in individuals with a personal or family history of medullary thyroid carcinoma or multiple endocrine neoplasia type 2 syndrome [80]. This precaution is based on preclinical studies demonstrating GLP-1 receptor agonists-mediated stimulation of thyroid parafollicular C-cells, leading to calcitonin secretion and C-cell proliferation in rodent models [81]. Although GLP-1 receptor agonists expression appears limited in human thyroid tissue [82], patients with MEN2 harbor germline RET mutations that predispose to C-cell tumorigenesis [83]. Accordingly, regulatory agencies recommend avoiding GLP-1-based therapies, including tirzepatide, in these populations [84].

Additional adverse events reported include peripheral neuropathy (e.g., peroneal nerve palsy associated with rapid weight loss), alopecia, hypersensitivity reactions, cholelithiasis, cholecystitis, and injection-site reactions [76,85,86,87,88]. There have also been concerns regarding potential thyroid tumor risk, although further evidence is required to clarify this association [87].

In patients with OSA, who are frequently multimorbid, the introduction of an additional long-term pharmacological therapy requires careful clinical evaluation of risks and benefits. Significant weight loss may also be associated with reductions in lean muscle mass and bone density, particularly in older adults [89]. In cases of insufficient therapeutic response (defined as <5% weight loss after three months at the full dose), discontinuation of treatment and consideration of alternative anti-obesity therapies is recommended, along with assessment of adherence and contributing factors [90].

Tirzepatide may also affect the absorption of oral medications, including hormonal contraceptives, due to delayed gastric emptying, particularly during dose escalation. This interaction may reduce the efficacy of oral contraceptives and should be considered in clinical practice [91].

## 9. Strengths and Limits of Actual Studies

The available literature on tirzepatide and its effects in OSA remains limited. Existing meta-analyses are constrained by heterogeneity among studies, including variations in treatment protocols, co-interventions, outcome measures, and follow-up durations [15]. Furthermore, most studies have focused on patients with moderate-to-severe OSA, limiting the generalizability of findings to individuals with mild disease.

Current analyses are primarily restricted to changes in AHI, body weight, and body mass index (BMI), with limited data available on other clinically relevant outcomes such as daytime sleepiness, metabolic parameters, and detailed polysomnographic measures. Additionally, the existing literature includes a greater emphasis on short-term efficacy and adverse events, with comparatively fewer studies evaluating cardiovascular outcomes [15].

Long-term safety data for tirzepatide are still emerging. Gastrointestinal adverse events, including nausea, vomiting, and diarrhea, are commonly reported, and the high cost of therapy may limit accessibility and widespread clinical implementation. Further research is needed to evaluate the effects of tirzepatide on cardiovascular outcomes in patients with OSA, as well as its role in combination therapies with PAP or other weight-loss interventions [88].

Another important limitation is the potential for weight regain following treatment discontinuation. A systematic review and meta-analysis reported an average weight regain of approximately 9.69 kg after cessation of semaglutide or tirzepatide therapy [48,92], which may attenuate the metabolic and clinical benefits achieved during treatment.

Ongoing clinical trials are expected to provide additional data on the long-term safety, efficacy, cardiovascular outcomes, and cost-effectiveness of tirzepatide in the management of obesity, type 2 diabetes mellitus, and related conditions, including OSA [93].

## 10. Future Research Directions

A key unresolved question in the field is whether the improvement in OSA observed with tirzepatide is entirely attributable to weight loss or whether weight-independent mechanisms play a significant role. Clarifying this distinction will be essential for redefining therapeutic strategies and identifying patient subgroups most likely to benefit from incretin-based therapies [12,17].

Further studies are needed to evaluate the role of tirzepatide as part of combination or adjunctive treatment strategies, particularly in patients with incomplete response to established therapies such as upper airway surgery or mandibular advancement devices [94]. In addition, the potential use of emerging agents, such as oral nonpeptide GLP-1 receptor agonists (e.g., orforglipron), to maintain weight loss following tirzepatide therapy warrants investigation [95,96].

An important clinical question is whether incretin-based therapies could complement or, in selected cases, challenge continuous PAP as first-line treatment in patients with OSA and comorbid obesity. However, direct head-to-head comparative trials are required, focusing on outcomes such as AHI, daytime sleepiness, sleep-related quality of life, and blood pressure [13,19].

Long-term studies are also necessary to determine whether the benefits observed in the SURMOUNT-OSA trials are sustained over time and to assess their impact on hard cardiovascular outcomes, including myocardial infarction and stroke [97]. In addition, further investigation is required to determine the extent to which weight loss alone explains the observed improvements in OSA, or whether direct effects on respiratory physiology are involved [17].

Potential adverse consequences of substantial weight loss, such as loss of skeletal muscle mass and the development of sarcopenia, should also be considered, particularly in older adults [98]. In this context, adjunctive therapies such as bimagrumab or selective androgen receptor modulators are being explored to preserve or enhance muscle mass during weight reduction [99,100].

Future research should also include long-term trials comparing tirzepatide with other incretin-based therapies, such as semaglutide, with particular emphasis on durability of weight loss, cardiometabolic outcomes, and patient-reported quality of life [65]. Given the increasing use of pharmacological treatments for obesity, further evaluation of cost-effectiveness and real-world implementation is warranted. Prescription rates for obesity medications continue to rise, reflecting growing clinical adoption and highlighting the need for sustainable and equitable treatment strategies [101].

Overall, a comprehensive research approach integrating mechanistic, clinical, and health system perspectives will be essential to determine the full therapeutic potential of tirzepatide in OSA and related metabolic disorders.

## 11. Future Considerations Regarding the Mission of Pulmonologists

Pulmonologists should play an integral role within multidisciplinary teams managing patients with OSA and obesity. A more holistic approach to care is required, incorporating both pharmacological and lifestyle interventions, as well as patient education regarding weight management and treatment adherence. In addition, careful consideration should be given to treatment discontinuation, particularly in the context of adverse effects or disparities in access to therapy [102].

Combined treatment strategies targeting both OSA and obesity may provide greater clinical benefit than addressing either condition in isolation. The concomitant use of CPAP and tirzepatide has been associated with improvements in cardiometabolic parameters, including blood pressure, triglyceride levels, and insulin sensitivity [103]. These findings highlight the importance of integrating pulmonologists into multidisciplinary care pathways to optimize patient outcomes.

## 12. Conclusions

Tirzepatide represents a significant advance in the management of obesity and its related comorbidities, including obstructive sleep apnea. Current evidence demonstrates substantial improvements in OSA severity, largely attributed to weight reduction. However, emerging data suggest that additional mechanisms—such as modulation of inflammation, metabolic regulation, and possibly ventilatory control—may contribute to these effects. These findings raise the possibility that tirzepatide may extend beyond a symptomatic or adjunctive therapy and instead represent a step toward disease modification in obesity-related OSA. Nevertheless, definitive evidence supporting weight-independent effects remains limited, and further mechanistic and long-term studies are required.

The integration of pharmacological therapies such as tirzepatide into OSA management may signal a transition from purely device-based approaches toward more comprehensive, pathophysiology-driven strategies. Future research should focus on comparative effectiveness with established treatments such as CPAP, identification of optimal patient phenotypes, and evaluation of long-term clinical outcomes, including cardiovascular risk reduction.

Ultimately, while tirzepatide does not replace established therapies such as CPAP, it has the potential to redefine the management of OSA by targeting its underlying metabolic drivers, supporting a shift toward integrated, pathophysiology-based treatment strategies.

## Figures and Tables

**Table 1:** Summary of clinical evidence on tirzepatide in obesity and obstructive sleep apnea, stratified by relevance to OSA-specific outcomes.

Study/Trial	Population	Intervention	Duration	Key Outcomes	Main Findings	Relevance to OSA
SURMOUNT-1 [38]	Obesity (no T2DM, no OSA)	Tirzepatide 5–15 mg	72 weeks	Weight loss	Induced substantial and sustained weight reduction (−19.5% to −20.9%) compared with placebo	Indirect (weight-loss/metabolic relevance)
SURMOUNT-OSA [33,35]	Obesity + OSA	Tirzepatide vs. placebo	52 weeks	AHI, PROs	Produced marked reductions in AHI (−20 to −25 events/h) and significant improvements in sleep-related outcomes	Direct (OSA-specific)
Bardóczi meta-analysis [15]	OSA patients	GLP-1RA/tirzepatide	≥12 weeks	AHI	Demonstrated a significant reduction in AHI (mean −14.45 events/h), supporting efficacy of incretin-based therapies in OSA	Direct (OSA-specific)
SURPASS trials [39,40]	T2DM	Tirzepatide vs. comparators	Various	HbA1c, weight	Showed superior glycemic control and clinically meaningful weight reduction across multiple phase 3 trials	Indirect (metabolic relevance)
SURMOUNT-5 [41]	Obesity (no OSA-specific population)	Tirzepatide vs. semaglutide	~72 weeks	Weight loss	Demonstrated greater weight reduction compared with semaglutide, reinforcing its high efficacy in obesity management	Indirect (weight-loss relevance)
Real-world study [36,37]	OSA + obesity	Tirzepatide vs. lifestyle	Observational	Mortality, CV events	Associated with reduced risks of all-cause mortality, major adverse cardiovascular events, and kidney outcomes	Indirect (OSA population, no AHI outcomes)

Abbreviations: OSA—obstructive sleep apnea; T2DM—type 2 diabetes mellitus; AHI—apnea–hypopnea index; PROs—patient-reported outcomes; HbA1c—Hemoglobin A1C. Direct evidence refers to studies including patients with obstructive sleep apnea and reporting sleep-specific outcomes (e.g., apnea–hypopnea index). Indirect evidence refers to studies conducted in populations without OSA or without reporting sleep-specific outcomes, but relevant through weight loss or metabolic effects.

## Data Availability

No new data were created or analyzed in this study. Data sharing is not applicable to this article, as it is based on previously published literature and publicly available sources.
